# Long-term outcomes of left bundle branch area pacing versus biventricular pacing in patients with heart failure and complete left bundle branch block

**DOI:** 10.1007/s00380-021-02016-5

**Published:** 2022-01-28

**Authors:** Juan Hua, Yang Chen, Jianhua Yu, Qinmei Xiong, Zhen Xia, Zirong Xia, Qianghui Huang, Qiling Kong, Huolong Chen, Yichu Zhang, Jianxin Hu, Juxiang Li, Jinzhu Hu, Qi Chen, Kui Hong

**Affiliations:** Department of Cardiology, The Second Affiliated Hospital of Nanchang University, 1 Minde Road, Nanchang, Jiangxi 330006 China

**Keywords:** Left bundle branch area pacing, Biventricular pacing, Heart failure, Complete left bundle branch block

## Abstract

Left bundle branch area pacing (LBBAP) has developed in an effort to improve cardiac resynchronization therapy (CRT). We aimed to compare the long-term clinical outcomes between LBBAP and biventricular pacing (BIVP) in patients with heart failure (HF) and complete left bundle branch block (CLBBB). Consecutive patients with HF and CLBBB requiring CRT received either LBBAP or BIVP were recruited at the Second Affiliated Hospital of Nanchang University from February 2018 to May 2019. We assessed their implant parameters, electrocardiogram (ECG), clinical outcomes at implant and during follow-up at 1, 3, 6, 12, and 24 months. Forty-one patients recruited including 21 for LBBAP and 20 for BIVP. Mean follow-up duration was 23.71 ± 4.44 months. LBBAP produced lower pacing thresholds, shorter procedure time and fluoroscopy duration compared to BIVP. The QRS duration was significantly narrower after LBBAP than BIVP (129.29 ± 31.46 vs. 156.85 ± 26.37 ms, *p* = 0.005). Notably, both LBBAP and BIVP significantly improved LVEF, LVEDD, NYHA class, and BNP compared with baseline. However, LBBAP significantly lowered BNP compared with BIVP (416.69 ± 411.39 vs. 96.07 ± 788.71 pg/ml, *p* = 0.007) from baseline to 24-month follow-up. Moreover, patients who received LBBAP exhibited lower number of hospitalizations than those in the BIVP group (*p* = 0.019). In addition, we found that patients with moderately prolonged left ventricular activation time (LVAT) and QRS notching in limb leads in baseline ECG respond better to LBBAP for CLBBB correction. LBBAP might be a relative safe and effective resynchronization therapy and as a supplement to BIVP for patients with HF and CLBBB.

## Introduction

Heart failure (HF) is a major public health issue with high morbidity and mortality, resulting in considerable financial and service burdens to the health system [1]. Left bundle branch block (LBBB) causes dyssynchronous electrical activation of the heart and creates discoordinate contraction of the left ventricle (LV), which leads to or aggravates HF [2]. Cardiac resynchronization therapy (CRT) using biventricular pacing (BIVP) has been recommended to improve cardiac functionality and enhance prognosis of patients with advanced HF when the optimal drug treatment still fails to improve the symptoms of HF [3]. However, the procedure for implanting the LV pacing lead of BIVP is quite complex, particularly in patients with venous malformations or coronary vein stenoses [4–6]. Furthermore, approximately 30% of patients have nonresponse to BIVP [7, 8]. His-Purkinje system pacing is currently considered the optimal physiologic pacing method with the pacing lead directly implanted in the conduction system to narrow QRS wave and improve cardiac function by selective or nonselective His-bundle pacing (HBP) [9, 10]. Nevertheless, HBP has several shortcomings limiting its application, such as a relatively lower success rate, high corrective threshold and low R-wave amplitude due to the specific anatomic characteristics of the His bundle [11]. In addition, HBP implantation may easily injure the bundle branch and exacerbate occurrence of atrioventricular (AV) block [12–14].

Huang et al. [15] first presented left bundle branch pacing (LBBP) in 2017, which targets pacing the proximal left bundle and its branches along with capture of LV septal myocardium. Selective LBBP (S-LBBP) only captures the LBB without myocardial capture, while nonselective LBBP (NS-LBBP) captures both the LBB and the local myocardium [16]. It is called LV septal endocardium pacing (LVSP) or deep septal pacing if only LV septal myocardium is captured [16]. Left bundle branch area pacing (LBBAP), with the lead implanted slightly distal to the His bundle and screwed deep in the LV septum ideally to capture LBB according to the ESC guidelines in 2021 [17], means LBBP or LVSP, without clear evidence for LBB capture [18]. Accumulating studies have shown that LBBAP can correct complete left bundle branch block (CLBBB), restore LV synchrony in HF patients, and improve cardiac function as well as symptoms in these patients, but the average period of follow-up for these studies was relatively short ranging from 6 to 12 months [15, 19, 20]. Therefore, we aimed to prospectively assess the long-term effects and safety in patients with HF and CLBBB after LBBAP and BIVP.

## Methods

### Patient recruitment

**Fig. 1:**
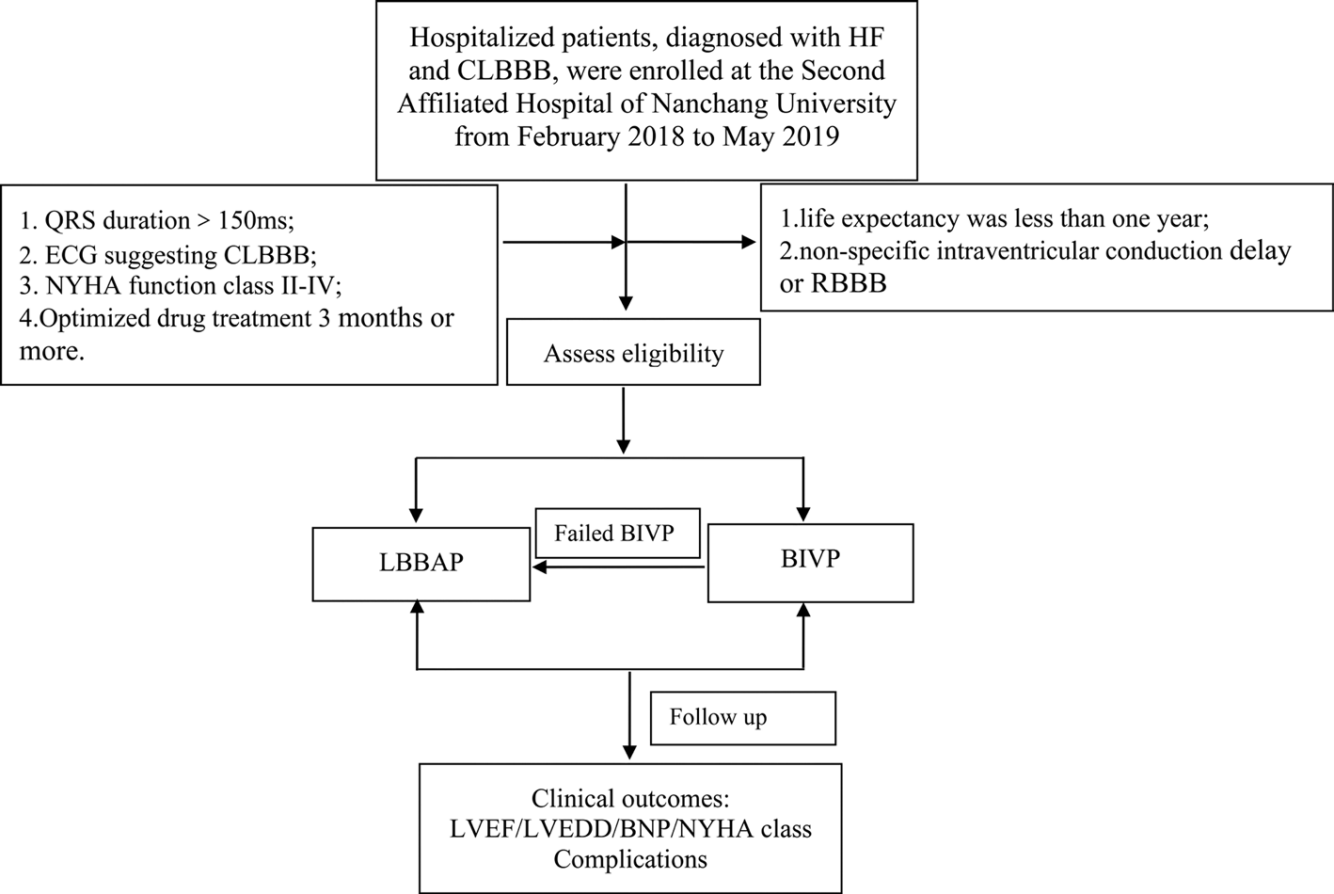
Schematic diagram of the study. *LBBAP* left bundle branch area pacing, *BIVP* biventricular pacing, *HF* heart failure, *CLBBB* complete left bundle branch block, *RBBB* right bundle branch block, *LVEF* left ventricular ejection fraction, *LVEDD* left ventricular end-diastolic diameter, *BNP* B-type natriuretic peptide, *NYHA* New York Heart Association

The hospitalized patients, diagnosed with HF and CLBBB, were enrolled at the Second Affiliated Hospital of Nanchang University from February 2018 to May 2019. The inclusion criteria were QRS duration (QRSd) > 150 ms, ECG suggesting traditional CLBBB, NYHA function class II–IV, and optimized drug treatment for 3 months or more. The exclusion criteria were life expectancy was less than 1 year, or those with non-specific intraventricular conduction delay or right bundle branch block (RBBB). LBBAP was an alternative choice to failed BIVP or first choice in place of a CS lead. This study was approved by the Ethics Committee of the Second Affiliated Hospital of Nanchang University. Each patient signed an informed consent prior to enrolment (Fig. 1).

### Study design

This was a single-center, non-randomized, prospective observational study, comprising patients with HF and CLBBB who were scheduled for pacing therapy and consecutively enrolled at our hospital. The baseline characteristics of enrolled patients are outlined in Table 1. All patients in this study were regularly followed-up at 1-, 3-, 6-, 12-, and 24-month post-implantation. A super response was defined as an increase in the LVEF to ≥ 50% at follow-up period. QRS duration was measured from the onset of QRS to the end of QRS during S-LBBP, and from the end of the stimulus artifact to the end of QRS during selective NS-LBBP and LVSP on lead V1.

**Table 1:** Baseline characteristics of patients who underwent LBBAP and BIVP

Parameters	LBBAP group (*n* = 21)		BIVP group (*n* = 20)	*p* value
Male, *n* %		15 (71.43)	15 (75.00)	0.796
Age, years		65.50 ± 6.91	67.50 ± 11.69	0.170
Medical comorbidities				
Hypertension, *n* %		6 (28.57)	11 (55.00)	0.086
Diabetes mellitus, *n* %		7 (33.33)	5 (25.00)	0.558
Renal dysfunction, *n* %		2 (9.52)	9 (45.00)	0.010
Atrial fibrillation, *n* %		5 (23.81)	4 (20.00)	0.768
HCM, *n* %		0	0	-
PCI, *n* %		1 (4.76)	3 (15.00)	0.269
QRSd, ms		177.91 ± 14.67	177.50 ± 16.99	0.935
Echocardiography parameters				
LVEF, %		30.05 ± 7.03	31.40 ± 9.30	0.601
LVEDD, mm		68.05 ± 10.30	66.60 ± 11.50	0.673
NYHA				
NYHA class II, *n* %		5 (23.81)	7 (35.00)	0.431
NYHA class III, *n* %		11 (52.38)	5 (25.00)	0.072
NYHA class IV, *n* %		5 (23.81)	8 (40.00)	0.265
NYHA class		3.00 ± 0.71	3.05 ± 0.89	0.824
BNP, pg/ml		851.65 ± 376.94	682.80 ± 821.39	0.041
Drug therapy				
ACEI/ARB/ARNI, *n* %		18 (85.71)	18 (90.00)	0.675
Beta-blocker, *n* %		18 (85.71)	17 (85.00)	0.948
Aldosterone antagonist, *n* %		19 (90.48)	18 (90.00)	0.959

*PCI* percutaneous coronary intervention, *HCM* hypertrophic cardiomyopathy, *LVEF* left ventricular ejection fraction, *LVEDD* left ventricular end-diastolic diameter, *NYHA* New York Heart Association, *BNP* B-type natriuretic peptide, *ACEI* angiotensin-converting enzyme inhibitor, *ARB* angiotensin II receptor blocker, *ARNI* angiotensin receptor-neprilysin inhibitor

### Implanting procedure

#### LBBAP

**Fig. 2:**
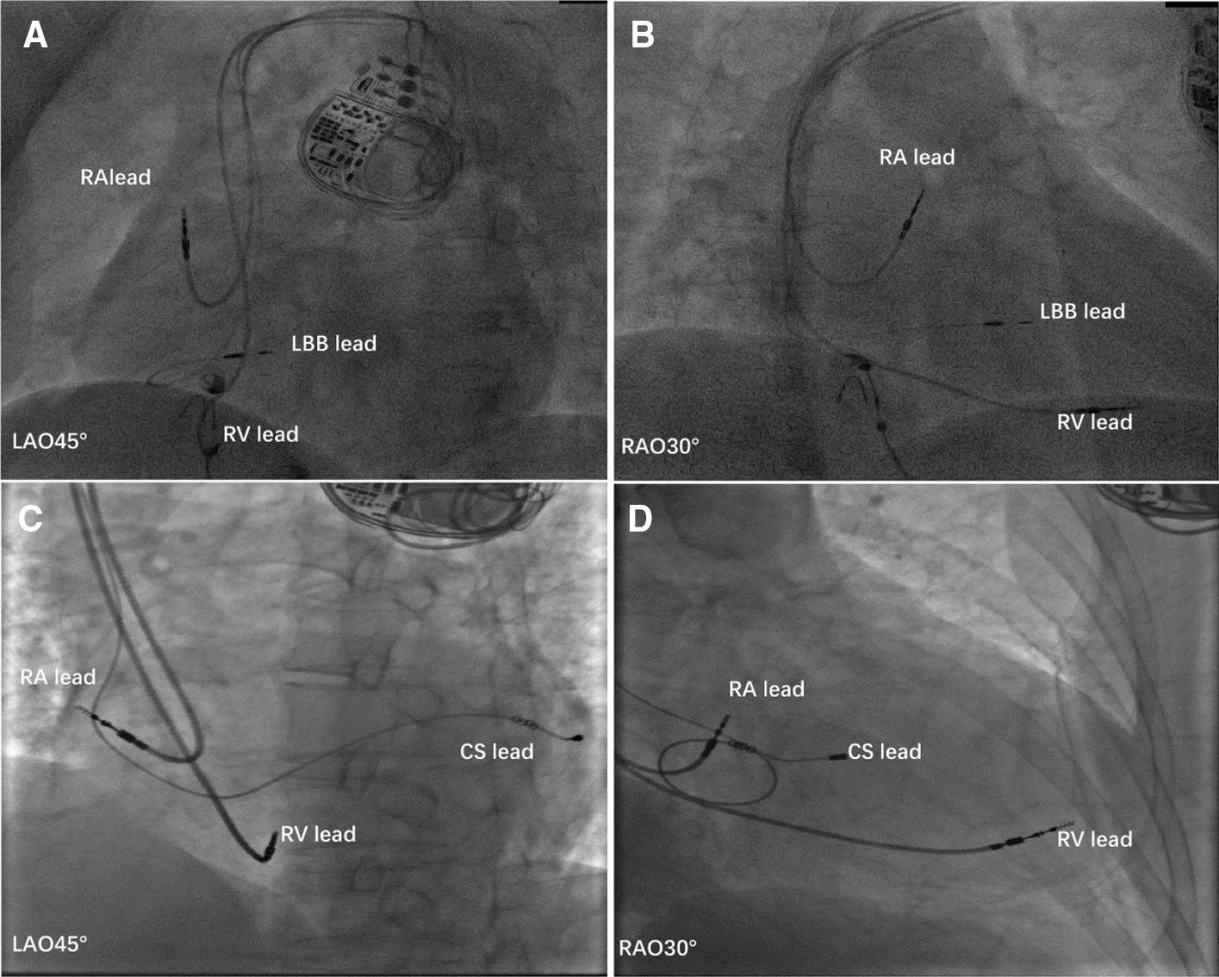
Representative images of cases from the LBBAP and BIVP groups. **A**, **B** Final images of the leads of LBBAP at LAO 45°and RAO 30°, respectively; **C**, **D** final images of leads of BIVP at LAO 45°and RAO 30°, respectively. *LBB* left bundle branch, *LV* left ventricular, *RV* right ventricular, *RA* right atrium, *CS* coronary sinus. Other abbreviations are as in Fig. 1

LBBAP was implemented using the Select Secure system (model 3830 lead, 69 cm; C315 His sheath, Medtronic, Inc., Minneapolis, MN) according to the methods described by Huang [21]. HBP was achieved and the image of distal HBP location was used to help determine the initial site for LBBP lead as previously reported [21]. Summarily, LBBAP was performed as follows: a 3830 lead was inserted through the C315 His sheath, they were advanced counter-clockwise in the ventricular apex direction (1–3 cm) to identify the ideal pacing site where the paced QRS complex presented a ‘W’ pattern in lead V1. The pacing lead was subsequently screwed towards the left side of the septum with a slight force for placement. Once ECG QRS morphology showed a pattern of RBBB or resembled normal QRS complex during pacing, and the unipolar pacing impedance of lead tip was not less than 500 ohms, the lead was considered to be at or near the left bundle branch and not penetrate the ventricular septal. At this point, the lead screwing was stopped. Finally, the pacing parameters were tested to confirm stable pacing threshold and consistent lead impedance, and the sheath removed (Fig. 2A, B).

The criteria of LBB capture followed by described previously [21]: (1) paced QRS morphology of RBBB pattern in lead V1; (2) the stimulus to peak left ventricular activation time (stim-LVAT, defined as stimulus to peak R-wave in lead V6) shortens abruptly with increasing output and remains shortest and constant at different outputs. Successful LBBAP was considered to be met both the criteria above mentioned. However, it was considered LV septal capture if none of above criteria was met. The characteristics of the ECG and the intracardiac electrogram (EGM) were observed and used to distinguish LBBP from LVSP. S-LBBP results a typical RBBB morphology with a discrete component between the pacing stimulus and ventricular activation in the EGMs, while NS-LBBP results in a narrow RBBB morphology without the discrete component. However, LVSP results in a RBBB morphology in some cases without the discrete component [18, 19]. The criteria of the correction of CLBBB by LBBAP was characterized by followings: (1) CLBBB morphology disappeared, and paced QRS morphology of RBBB pattern in lead V1 and QRS duration became narrow (full correction of the CLBBB with a paced QRSd ≤ 130 ms [22]); (2) short LVAT; 3) The position of lead tip was under the sub-endocardium of IVS [23].

#### BIVP

For BIVP implantation [24], the right ventricular lead was positioned in the RV apex and a coronary sinus (CS) lead positioned in the most clinically suitable lateral ventricular branch. The right-atrial (RA) lead was implanted at the RA appendage. The LV lead was implanted in the posterolateral or anterolateral vein by standard operating process, whereas the AV sequential pacing for biventricular pacing was performed via devices (Fig. 2C, D).

### Data collection

We recorded fluoroscopy duration for LBBAP lead implantation and procedure time at implant. Lead parameters, including unipolar tip pacing thresholds and impedances of LBBAP or BIVP, were measured at implant and during regular follow-up of 1-, 3-, 6-, 12-, and 24-month. We measured QRS duration at implant and follow-up. We obtained echocardiography (LVEF and LVEDD) at preimplantation and regular follow-up of 1-, 6-, 12-, and 24-month. We also documented BNP concentration and NYHA class, and tracked complications and clinical outcomes, such as death and rehospitalization, during follow-up.

### Statistical methods

All statistical analyses were performed using SPSS software version 24.0. Continuous variables were presented as means ± standard deviations (SDs), and compared using two-tailed Student’s *t*- or rank-sum tests. On the other hand, categorical data were presented as numbers and percentages, and analyzed using the chi-squared or Fisher's exact tests. Data followed by *p* < 0.05 were considered statistically significant.

## Results

### Baseline characteristics

Clinical characteristics of all included patients are summarized in Table 1. Summarily, a total of 41 hospitalized patients with HF and CLBBB were enrolled and followed-up for a mean duration of 23.71 ± 4.44 months, of which 21 underwent LBBAP (mean 65.50 ± 6.91 years, 71.43% males) while 20 received BIVP (mean 67.50 ± 11.69 years, 75% males). Nineteen patients underwent LBBAP as the first-line strategy, and rescue LBBAP were performed in two patients with failed CS-LV lead implantation. Of the participants in the LBBAP group, 61.9% were selective LBBP (13/21), 33.3% were nonselective LBBP (7/21), and 4.8% were LVSP (1/21). Patients in the LBBAP group had a higher baseline BNP than those in the BIVP group (851.65 ± 376.94 vs. 682.80 ± 821.39 pg/ml, *p* = 0.041), but a lower incidence of chronic renal insufficiency (9.52% vs. 45.00%, *p* = 0.010). We observed no significant differences between the two groups with regards to the other clinical characteristics (Table 1). All patients were treated with optimized drug therapy, and were followed-up at 1-, 3-, 6-, 12-, and 24-month after pacing.

### Procedural pacing parameters in patients with successful LBBAP and BIVP

**Fig. 3:**
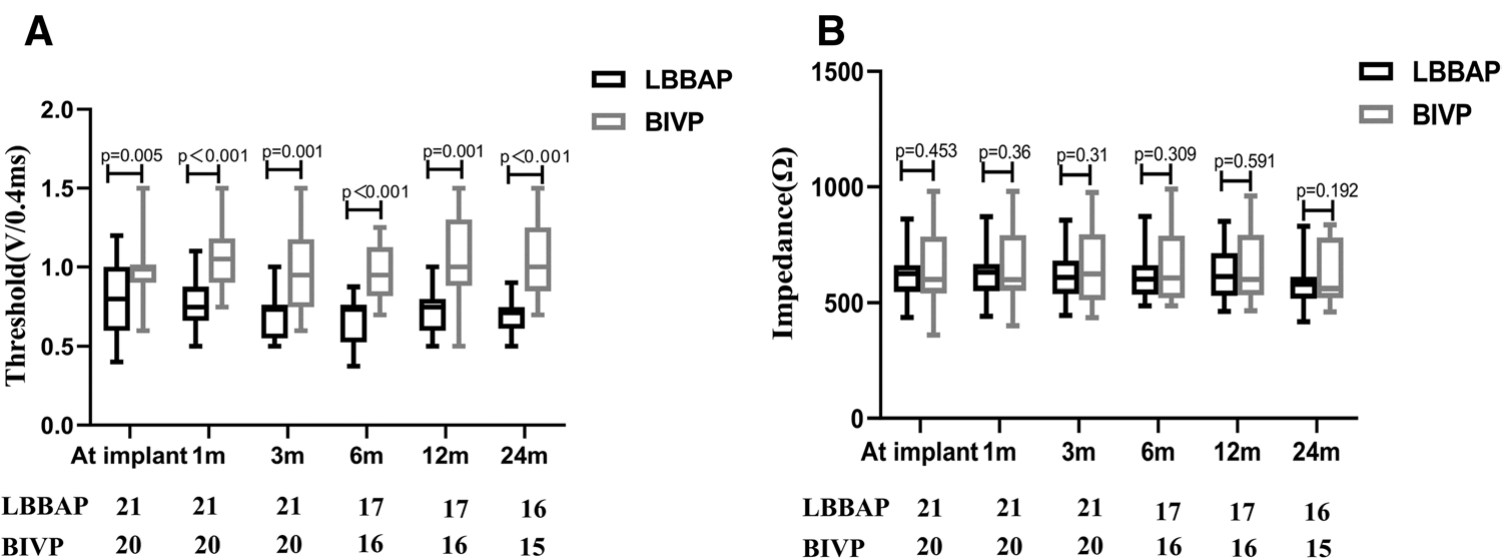
Pacing parameters of LBBAP and BIVP. **A** LV or 3830 lead pacing thresholds of patients. **B** Pacing impedance of patients. The number below the picture represents the number of patients at each point of follow-up. Abbreviations are as in Figs. 1 and 2

Comparisons in pacing parameters between patients in the LBBAP and BIVP groups are summarized in Table 2. Summarily, LBBAP resulted in significantly shorter procedural time (104.24 ± 17.36 vs. 127.80 ± 24.71 min, *p* = 0.001) and fluoroscopy duration (20.14 ± 6.05 vs. 26.50 ± 4.07 min, *p* = 0.011) than BIVP. Additionally, capture thresholds of 3830 lead of LBBAP were lower than those of LV lead of BIVP at implantation, with these differences found to persist at 1-, 3-, 6-, 12-, and 24-monthduring follow-up (Table 2). However, we found no significant differences in pacing impedance between the LBBAP and BIVP groups at both implantation and follow-up (Table 2). Specifically, pacing thresholds of patients in the LBBAP group remained stable at 1-, 3-, 6-, 12-, and 24-month of follow-up (0.76 ± 0.15 vs. 0.69 ± 0.15 vs. 0.66 ± 0.15 vs. 0.72 ± 0.16 vs. 0.70 ± 0.10 V at 0.4 ms) (Fig. 3A), but pacing impedances of LBBAP decreased slightly during follow-up (625.5 ± 115.59 vs. 617.69 ± 112.23 vs. 622.25 ± 105.13 vs. 629.00 ± 121.52 vs. 575.81 ± 95.75 Ω) (Fig. 3B).

**Table 2:** Pacing parameters of patients in the LBBAP and BIVP groups

Procedural characteristics	LBBAP group (*n* = 21)	BIVP group (*n* = 20)	*p* value
Pacing types, *n*%			
DDDR	6(28.57)	0(0)	0.010
ICD (single‐chamber)	1(4.76)	0(0)	0.323
ICD (dual‐chamber)	2(9.52)	0(0)	0.157
CRT-P	9(42.85)	13(65.00)	0.155
CRT-D	3(14.30)	7(35.00)	0.123
Procedural time, min	104.24 ± 17.36	127.80 ± 24.71	0.001
Fluoroscopy duration, min	20.14 ± 6.05	26.50 ± 4.07	0.011
R-wave amplitude, mV	8.62 ± 3.18	9.82 ± 2.56	0.120
Pacing parameters			
LV or 3830 lead impedance, Ω			
At implant	621.94 ± 114.6	654.63 ± 179.01	0.453
1-month follow-up	625.5 ± 115.59	661.81 ± 174.17	0.360
3-month follow-up	617.69 ± 112.23	669.75 ± 173.86	0.310
6-month follow-up	622.25 ± 105.13	667.31 ± 165.99	0.309
12-month follow-up	629.00 ± 121.52	651.12 ± 154.53	0.591
24-month follow-up	575.81 ± 95.75	632.88 ± 140.87	0.192
LV or 3830 lead thresholds, at 0.4 ms, V			
At implant	0.78 ± 0.22	1.03 ± 0.30	0.005
1-month follow-up	0.76 ± 0.15	1.05 ± 0.21	< 0.001
3-month follow-up	0.69 ± 0.15	1.03 ± 0.44	0.001
6-month follow-up	0.66 ± 0.15	0.98 ± 0.18	< 0.001
12-month follow-up	0.72 ± 0.16	1.04 ± 0.27	0.001
24-month follow-up	0.70 ± 0.10	1.16 ± 0.42	< 0.001

### The 12-ECG from LBBAP and BIVP and changes in QRS duration

**Fig. 4:**
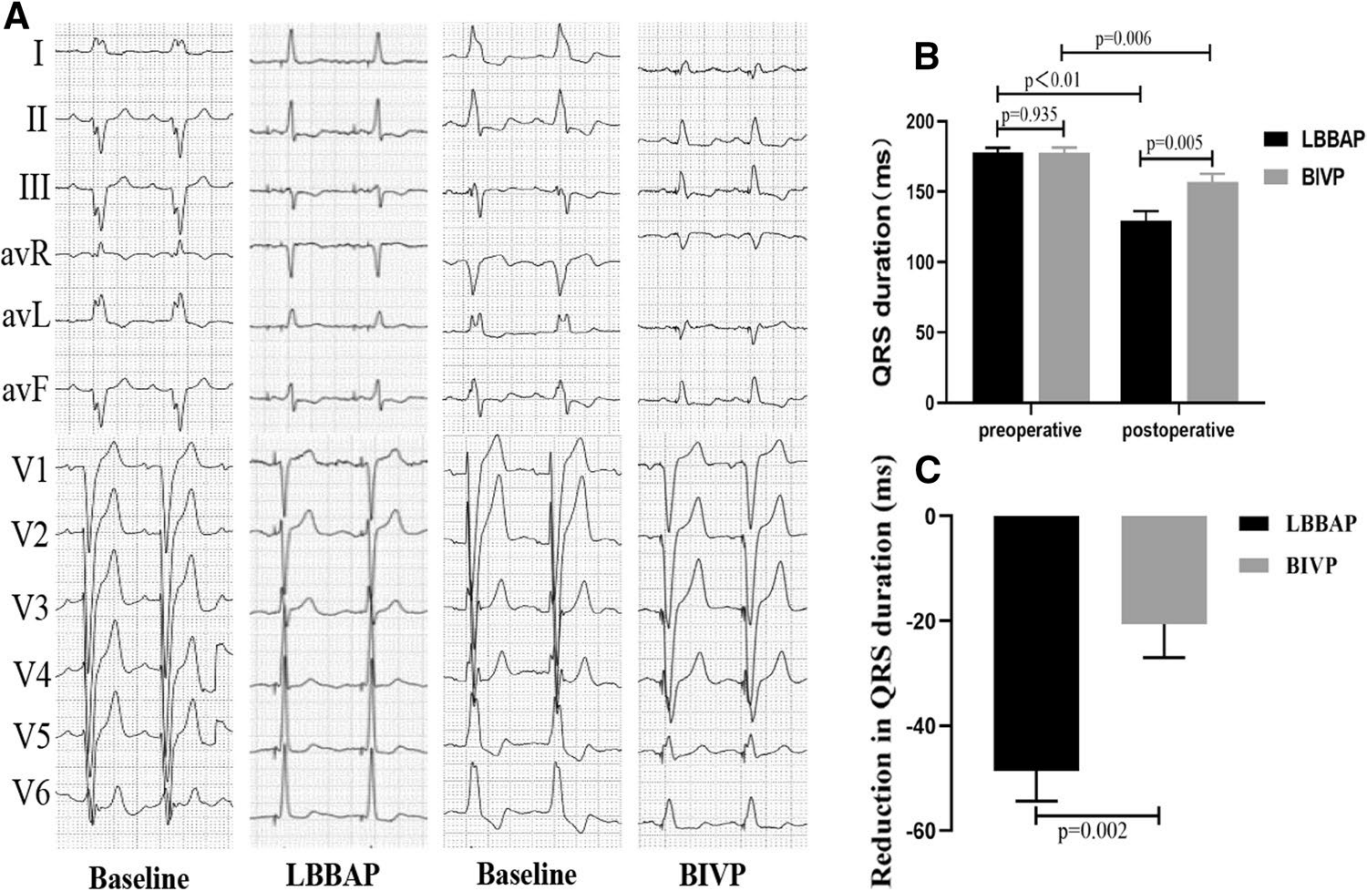
LBBAP or BIVP corrected CLBBB in HF patients. Twelve-lead ECG from LBBAP and BIVP are illustrated. **A** Morphology of QRS for sinus rhythm (s) and pacing (p) in patients who received LBBAP and BIVP, respectively. LBBAP shortened QRS duration from 172 to 120 ms, whereas baseline QRS width from 165 to 143 ms in BIVP. **B** ECG significantly shortened QRS duration after LBBAP or BIVP. **C** LBBAP significantly lowered QRS duration compared to BIVP. Abbreviations are as in Fig. 1

A comparison of QRS duration (QRSd) between baseline and follow-up after LBBAP or BIVP revealed significantly narrowing of the 12-lead ECG QRSd in both groups (Fig. 4A). In the LBBAP group, QRSd of the 21 patients significantly decreased from a baseline 177.91 ± 14.67 to 129.29 ± 31.46 ms (*p* < 0.001), while those of the 20 patients in the BIVP group significantly decreased from baseline 177.50 ± 16.99 to 156.85 ± 26.37 ms (*p* = 0.006) (Fig. 4B). Furthermore, patients in the LBBAP group recorded a significantly narrower mean paced QRSd than those in the BIVP group (129.29 ± 31.46 vs. 156.85 ± 26.37 ms, *p* = 0.005, paced QRSd ≤ 130 ms in 13 patients of LBBAP group). Consistent with the above results, LBBAP resulted in a greater reduction in QRS duration compared with BIVP (48.62 ± 26.29 vs. 20.65 ± 28.28 ms, *p* = 0.002) (Fig. 4C).

### Evaluation of cardiac function in patients with LBBAP or BIVP

**Fig. 5:**
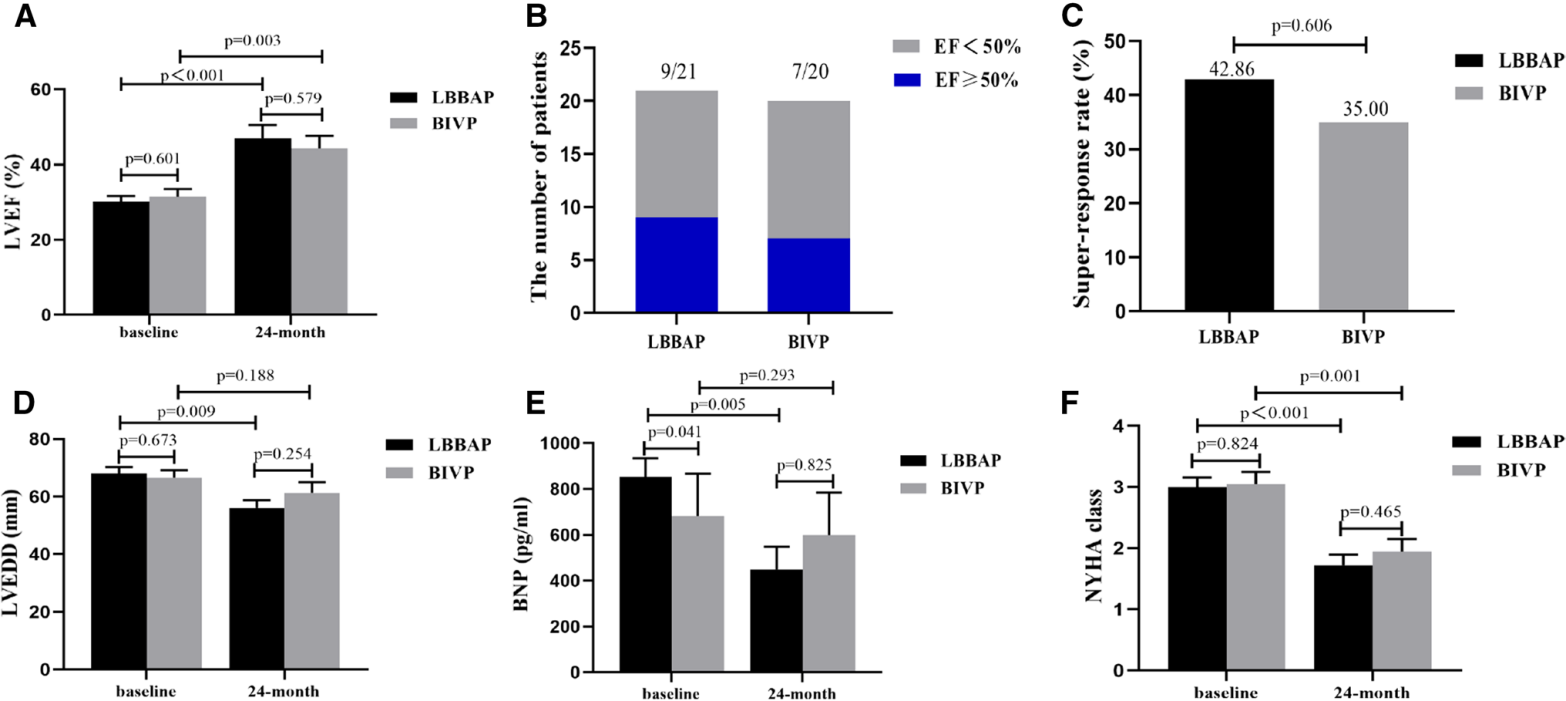
Clinical outcomes at baseline and 24-month follow-up after LBBAP or BIVP. **A** LBBAP or BIVP improved LVEF; **B**, **C** there was no significant difference in super-response between LBBAP and BIVP groups; **D**, **E** LBBAP had a significant reduction in LVEDD and BNP, while no significant differences of LVEDD and BNP was observed in BIVP group; **F** NYHA class improved in both groups. Abbreviations are as in Fig. 1

Comparisons in LVEF, super-response rate, LVEDD, BNP level and NYHA class between LBBAP and BIVP groups at baseline and 24-month follow-up are summarized in Fig. 3. Notably, patients in both groups exhibited significantly elevated LVEF in both LBBAP and BIVP from baseline to follow-up of 24-month (30.05 ± 7.03% to 47.00 ± 14.90%, *p* < 0.001 and 31.40% ± 9.30 to 44.28 ± 14.26%, *p* = 0.003, respectively) (Fig. 5A). In addition, 9 out of 21 patients in the LBBAP group and 7 out of 20 patients in BIVP group exhibited super-response, although the super-response rate did not significantly differ between LBBAP and BIVP group (42.86% vs. 35.00%, *p* = 0.606) (Fig. 5B, C). Moreover, other cardiac function parameters, such as LVEDD (68.05 ± 10.30 to 56.06 ± 11.76 mm, *p* = 0.009), NYHA Class (3.00 ± 0.71 to 1.72 ± 0.75, *p* < 0.001) and BNP level (851.65 ± 376.94 to 449.66 ± 412.55 pg/ml, *p* = 0.005) were significantly lower in the LBBAP than BIVP group, and these were consistent with improvement in LVEF (Fig. 5D–F). On the other hand, patients who received BIVP also exhibited significantly improved NYHA functional class (3.05 ± 0.89 to 1.94 ± 0.87, *p* = 0.001) (Fig. 5F), although we found no significant differences in LVEDD and BNP between baseline and 24-month follow-up (66.60 ± 11.50 to 61.33 ± 15.63 mm, *p* = 0.188 and 682.80 ± 821.39 to 598.66 ± 783.75 pg/ml, *p* = 0.293, respectively) (Fig. 5D–E).

**Fig. 6:**
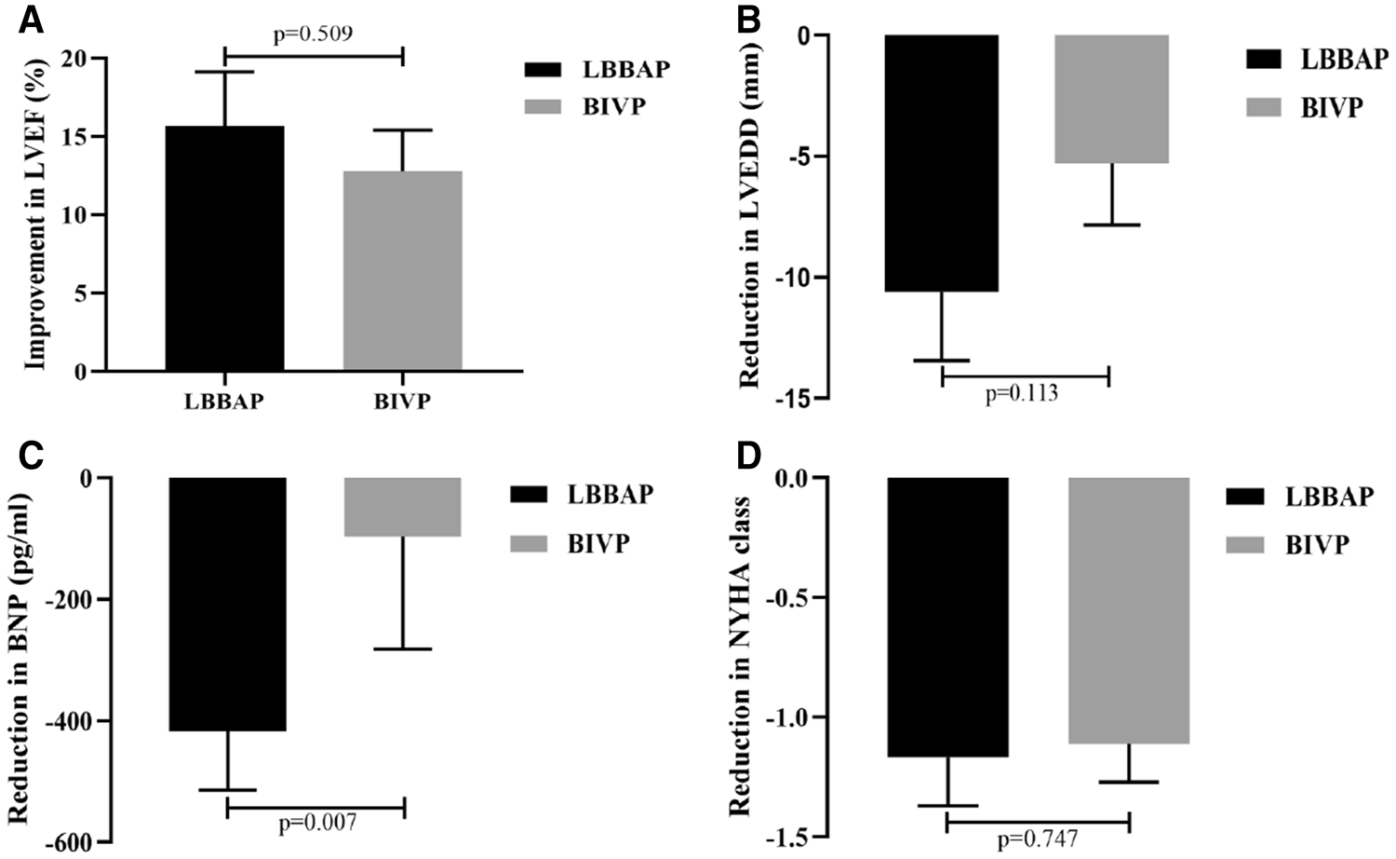
Comparison of changes in cardiac functional indicators between LBBAP and BIVP groups, after pacing at 24-month follow-up from baseline. **A** Improvement in LVEF with LBBAP and BIVP; **B** reduction in LVEDD with LBBAP and BIVP; **C** reduction in BNP with LBBAP and BIVP; **D** reduction in NYHA class with LBBAP and BIVP. Abbreviations are as in Fig. 1

We also compared cardiac function indicators between the LBBAP and BIVP groups, at 24-month follow-up, and found no significant differences in LVEF, LVEDD, BNP level, and NYHA class (Fig. 5A, D–F). A further comparison in changes in LVEF, LVEDD, BNP and NYHA class between the two groups revealed that the reduction in BNP was more significant in the LBBAP than the BIVP group (416.69 ± 411.39 vs. 96.07 ± 788.71 pg/ml, *p* = 0.007) (Fig. 6C). Moreover, LBBAP mediated a slight greater improvement in LVEF (15.66 ± 14.59% vs 12.77 ± 11.13%, *p* = 0.509), reduction in LVEDD (10.61 ± 11.97 vs 5.28 ± 10.81 mm, *p* = 0.113), and NYHA class (1.17 ± 0.86 vs 1.11 ± 0.68, *p* = 0.747) compared with BIVP at 24-month follow-up from baseline, although these changes were not statistically significant (Fig. 6A, B and D).

### CLBBB correction is associated with improved cardiac function

Among 21 patients, 13 achieved full correction of the CLBBB with a paced QRSd of ≤ 130 ms in the LBBAP group, while only 2 of 20 patients achieved a paced QRSd of ≤ 130 ms in the BIVP group. Obviously, the percentage of patients with a paced QRSd ≤ 130 ms was significantly higher in the LBBAP than BIVP group (13/21 vs 2/20, *p* = 0.001), indicating that LBBAP can achieve narrower QRSd compared with BIVP. Additionally, the rate of super response (LVEF ≥ 50%) in patients with full CLBBB correction (QRSd ≤ 130 ms) was significantly higher than that of patients with failed CLBBB correction (QRSd > 130 ms) in LBBAP group (8/13 vs 1/8, *p* = 0.027). Subgroup analysis suggested that full CLBBB correction was associated with improved LVEF.

**Fig. 7:**
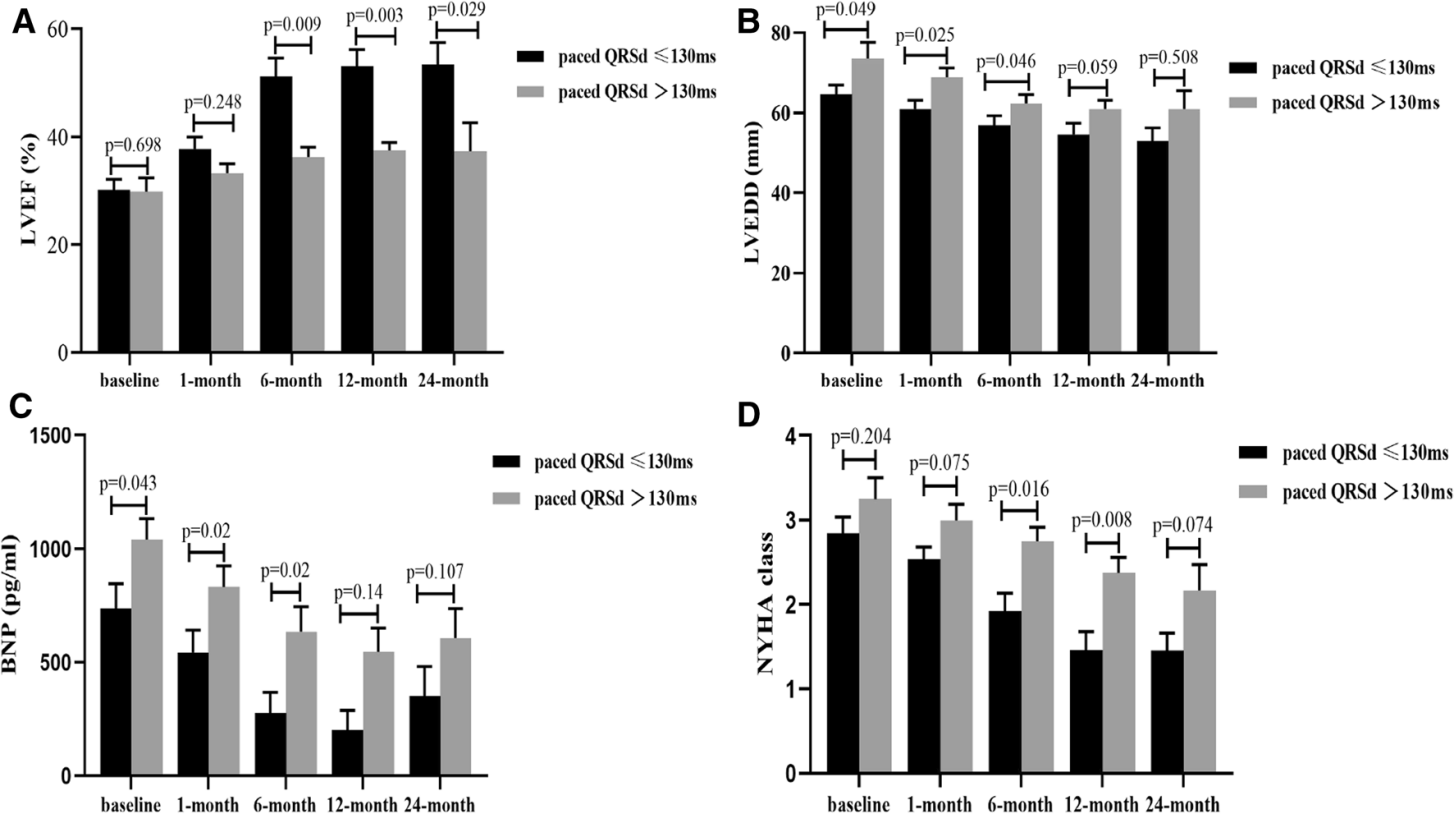
Correlation between QRS duration narrowing and improved cardiac function after LBBAP. Patients with CLBBB full correction achieved higher LVEF (**A**), lower LVEDD (**B**), lower BNP (**C**), and lower NYHA class (**D**) compared to those with failed CLBBB correction in the LBBAP group. Abbreviations are as in Fig. 1

Furthermore, we conducted a sub-analysis to compare the QRS duration between patients with full CLBBB correction and failed CLBBB correction after LBBAP. We found that the paced QRSd of full CLBBB correction was significantly shorter than that of failed CLBBB correction after LBBAP (108.77 ± 8.97 vs. 162.75 ± 25.08 ms, *p* < 0.001), while no significant difference between initial QRSd of CLBBB correction and failed CLBBB correction at baseline (176.00 ± 14.51 vs. 181.71 ± 15.38 ms, *p* = 0.430). Additionally, the sub-analysis at different follow-up periods, revealed that 13 patients with CLBBB correction exhibited higher LVEF, lower LEDD, lower BNP level and lower NYHA class compared to the 8 patients with failed CLBBB correction at 1-, 6-, 12-, and 24-monthfollow-up (Fig. 7A–D). These results further demonstrated that CLBBB correction was associated with improved cardiac function.

### The preoperative ECG characteristics and CLBBB correction

Based on the above results, we further analyzed ECG parameters of CLBBB patients in the LBBAP group at preimplantation. Interestingly, we found that preoperative 12-ECG lead V6 ventricular activation time (VAT) in patients with CLBBB correction was slightly shorter than that of patients with failed CLBBB correction (88.08 ± 23.90 vs 113.75 ± 35.03 ms, *p* = 0.06). In addition, we observed QRS notching in limb leads in 11 of 13 patients (84.62%) with CLBBB correction, while the notched R-wave occurred in only 2 of 8 patients (25.00%) with failed CLBBB correction (*p* = 0.018) (Table 3). No significant differences were found in the other preoperative ECG parameters between patients with full or failed CLBBB correction in the LBBAP group (Table 3).

**Table 3:** CLBBB full correction and ECG characteristics

ECG characteristics	Full CLBBB correction	Failed CLBBB correction	*p* value
Initial QRS width, ms	176.00 ± 14.51	181.71 ± 15.38	0.430
V6 VAT, ms	88.08 ± 23.90	113.75 ± 35.03	0.060
Q wave in lead I and AVL	6 (46.15%)	2 (25.00%)	0.400
QRS notch in lead V2-6	8 (61.54%)	3 (37.50%)	0.387
QRS notch in limb leads	11 (84.62%)	2 (25.00%)	0.018

*VAT *ventricular activation time

### Complications and clinical outcomes

We tracked complications and clinical outcomes, such as incidence of death and rehospitalization, in both groups during 24-month follow-up. Among 21 patients, 16 completed the follow-up of 24-month in the LBBAP group, while 15 out of 20 patients completed the whole follow-up in the BIVP group. Although no surgery-related complications occurred in both groups, one patient in each group died of HF (with LVEF lower than 35%). Four patients in the LBBAP group were re-admitted to the hospital because of worsening heart failure. On the other hand, one patient presented with symptoms of syncope because of being discharged after the pacemaker perceiving atrial fibrillation and was hospitalized twice. In the BIVP group, four patients were re-admitted to the hospital due to worsening heart failure, one was re-admitted three times, while another one patient (with LVEF less than 30%) was re-admitted six times. In total, the number of hospitalizations was significantly lower in the LBBAP than BIVP group (*p* = 0.019), which suggested that LBBAP significantly reduced the incidence of rehospitalization compared with BIVP.

## Discussion

In this study, we analyzed the clinical characteristics of patients with HF and CLBBB after LBBAP and BIVP, respectively, and further performed a detailed comparison on the effect of the approaches on ECG and cardiac function between them. Our major findings were as follows: (1) LBBAP is a feasible and safe approach for successful correction of CLBBB in patients with HF and CLBBB; (2) The long-term follow-up revealed that LBBAP significantly improved LVEF and NYHA functional class and further lowered BNP level and LVEDD; (3) LBBAP significantly shortened QRS duration and exerted better cardiac electrical resynchronization to relieve symptoms of HF, compared with BIVP; and (4) In the LBBAP group, patients with moderately prolonged LVAT and QRS with a notch in the limb leads in preoperative ECG, hence may benefit more from CLBBB full correction.

Multiple studies have shown that LBBAP exerts fewer perioperative complications with no fatal adverse effects [15, 25–28]. Results from our study revealed that neither procedural- nor device-related complications occurred in both groups, while LBBAP significantly reduced incidence of rehospitalization compared with BIVP. Our findings together with previous reports [25, 29] have indicated that LBBAP implanting is a relatively safe and effective procedure. Additionally, the capture thresholds of LBBAP were significantly lower than BIVP at implant, which was also significantly lower than that of HBP [25]. In fact, the LBBAP procedure had numerous advantages over BIVP, including shorter operation time and fluoroscopy duration, as well as lower and stable pacing thresholds. QRS duration is an established predictor of response to CRT [5], whereas its changes from preimplantation to post-implantation are also considered significant predictors of response to CRT [30]. Previous studies have demonstrated that LBBAP can significantly shorten the paced QRS duration [25, 31]. Results of the present study revealed significantly declined QRS durations in both LBBAP and BIVP groups, but the former were shorter than the latter. Additionally, LBBAP significantly improved LVEF, LVEDD, BNP and NYHA class at 24-month follow-up compared with baseline, while it also resulted in shorter QRS and lower BNP, than BIVP. Despite a lack of statistical significances, LBBAP caused a slight greater improvement in LVEF, as well as a greater reduction in LVEDD and NYHA class compared to BIVP. Collectively, these results demonstrated that LBBAP might be advantageous over BIVP in improving electrocardiographic and echocardiographic outcomes.

Although previous studies demonstrated that CRT therapy can significantly improve LVEF [32], only a few patients dramatically exhibited this effect, and have, therefore, been termed super-response [33]. We defined super-response as a final LVEF ≥ 50% at any point during follow-up [34]. The super-response rate observed in both groups in the present study was slightly lower than that previously reported [29, 35]. Additionally, the percentage of patients with a QRS less than 130 ms was significantly higher in the LBBAP than BIVP group, although the rate of super response was similar between the groups. The seemly paradoxical result may be due to differences in the mechanisms of LBBAP and BIVP therapy. Particularly, LBBAP directly paces the left bundle branch bypassing the block region, thereby generating a physiological cardiac conduction by synchronizing delayed LV activation and intrinsic activation in the right ventricle [19, 29]. In contrast, BIVP paces two non-physiological sides in the ventricles for resynchronization between the left and right ventricle, thereby prolonging LV activation time [36, 37]. Consequently, LBBAP can shorten QRS duration and significantly improve LVEF compared to BIVP. Another possible reason may be due to the small sample size of patients and the non-randomized study design.

Previous studies have reported successful correction of CLBBB by LBBAP, with high success rates achieved [20, 38]. For example, Vijayaraman et al. [35] reported that LBBAP resulted in a high success rate (88%) of CLBBB correction in patients. By comparison, our study obtained a lower success rate (61.90%) for CLBBB correction, partly due to operator experience at the early stage. In addition, some CLBBB patterns, such as left ventricular slow conduction caused by myocardial lesions and scar or conduction disorder caused by distal branch of left bundle branch, cannot be completely corrected using His-Purkinje-mediated ventricular activation [39]. We performed subgroup analysis based on CLBBB correction or failed correction by LBBAP, and found that patients with CLBBB correction exhibited significantly improved cardiac functions, such as LVEF, LVEDD, BNP, and NYHA class, compared with those with failed CLBBB correction. These results, together with those from a recent study [39], further demonstrated that correction of CLBBB is associated with improved cardiac function in patients after LBBAP. A possible explanation for this is that correction of CLBBB by LBBAP restores electrical and mechanical synchrony of the left ventricle. Taken together, these findings indicate that LBBAP significantly improves echocardiographic and clinical parameters compared with BIVP.

As previously discussed, LBBAP might be advantageous over BIVP in treating patients with HF and CLBBB. We attempted to explore which patients with HF are best suited for LBBAP. Interestingly, we found that the preoperative 12-ECG lead V6 ventricular activation time (VAT) in patients with CLBBB correction was significantly shorter than in those that with failed CLBBB correction. Our results are consistent with previous studies which have reported that patients with CLBBB exhibited LVAT greater than 60 ms in leads V5 and V6 [40, 41], indicating that prolonged LVAT may be an indicator of CLBBB. However, we observed that V6 VAT in patients with CLBBB correction was slightly shorter than that of patients with failed CLBBB correction by LBBAP, suggesting that patients with moderately prolonged LVAT in preoperative ECG are hence more easily to achieve CLBBB correction. Therefore, we speculate that moderately prolonged LVAT implies the presence of a block site in the proximal part of left bundle branch, hence LBBAP can immediately cross the conduction block site and effectively correct CLBBB. In contrast, cases with significantly prolonged LVAT may partly result from the conduction block site of distal part of left bundle branch or left ventricular conduction delay caused by myocardial lesion itself or myocardial scar, which can be failed to be corrected by LBBAP. We also found that the rate of full CLBBB correction in patients with QRS with a notch in preoperative limb leads was significantly higher than that of patients with no QRS with a notch in limb leads after pacing. A recent study indicated that about 1/3 of all LBBB cases, diagnosed by conventional criteria, may be a combination of LV hypertrophy and left anterior fascicular block, and not true CLBBB [42]. Moreover, Wagner et al. [42] proposed that true CLBBB had longer QRS duration and mid-QRS notching. Notches in limb leads represents the time when the electrical depolarization wave front reaches the endocardium of the LV and the epicardium of the posterolateral wall [42], indicating that notch in leads is an indicator of true CLBBB. Our results demonstrated that patients with moderately prolonged LVAT and mid-QRS notching in the limb leads in preoperative ECG could easily achieve full CLBBB correction with LBBAP. Taken together, these results showed that patients with heart failure and CLBBB, particularly those with moderately prolonged LVAT and QRS notch in limb leads, respond better to LBBAP, thereby exhibiting beneficial clinical outcomes.

There have been some articles published about the comparisons between LBBAP and BIVP [23, 43], which have demonstrated that LBBAP is an alternative method to BIVP in CRT treatment, our results were consistent with them. Compared with these studies, we have some advantages: (1) We had a longer duration of follow-up for 24-month; (2) We performed subgroup analyses by CLBBB correction and found that patients with CLBBB correction may get a better clinical outcome compared with those who with failed CLBBB correction; (3) We tried to analysis which type of patients would respond better to LBBAP through scanning baseline ECG characteristics. However, more larger sample size researches are needed to verify our conjecture.

## Limitations

There were several limitations in the present study. First, the small number of patients and non-randomized design may lead to inadequate power. LBBAP was in its early phase of clinical application in our hospital, and was only used in a small size of patients. Second, the definition of strict criteria for LBB capture was not used in the study. The characteristics of the ECG and the EGM in the LBBAP procedure, such as stim-LVAT, paced QRS morphology, and discrete component in the EGM, as the indirect criteria for LBB capture, were mainly used to distinguish LBBP from LVSP in this study. Indeed, it was difficult to distinguish them accurately in some cases. Currently, Wu et al. [16] proposed that retrograde His potential on the HBP lead and/or anterograde left conduction system potentials on the multielectrode catheter during LBBP were defined as the criteria for direct LBB capture, which could be used to distinguish LBBP from LVSP more accurately. Furthermore, the morphological characteristics of CLBBB in our study were not strictly met Strauss's criteria, which may lead to an underestimation of the efficacy of LBBAP compared to BIVP for CRT. Moreover, it was clear that the number of patients with renal dysfunction was significantly lower in the LBBAP group than that of the BIVP group, which may affect the lower hospitalization rate and mortality rate of LBBAP compared with BIVP. Since the renal dysfunction was recognized as a risk factor for higher HF hospitalization and all-cause mortality [44]. Collectively, we initially compared efficacy and clinical benefits of patients with HF and CLBBB, between LBBAP and BIVP groups, for 24-month follow-up. However, this was a small observational study with possible selection bias. Therefore, further studies using larger sample sizes are required to validate these findings.

## Conclusions

In summary, LBBAP can significantly synchronize LV activation by correcting CLBBB and promote LV reverse remodeling. LBBAP is more effective in shortening QRS duration and enhancing echocardiographic and clinical responses than BIVP, therefore, may be a better candidate for resynchronization therapy for patients with HF and CLBBB.
